# Modification of stool's water content in constipated infants: management with an adapted infant formula

**DOI:** 10.1186/1475-2891-10-55

**Published:** 2011-05-19

**Authors:** Dámaso D Infante, Oscar O Segarra, Susana S Redecillas, Marina M Alvarez, Mar M Miserachs

**Affiliations:** 1 1Unit of Paediatric Gastroenterology, Hepatology and Nutrition. Hospital Universitari Vall d'Hebron, Universitat Autònoma de Barcelona, Barcelona, Spain

**Keywords:** constipation, infant, NIRA, lactose, magnesium

## Abstract

**Background:**

Constipation is a common occurrence in formula-fed infants. The aim of this preliminary study was to evaluate the impact of a formula with high levels of lactose and magnesium, in compliance with the official regulations, on stool water content, as well as a parental assessment of constipation.

**Materials and methods:**

Thirty healthy term-born, formula-fed infants, aged 4-10 weeks, with functional constipation were included. All infants were full-term and fed standard formula. Exclusion criteria were preterm and/or low birth weight, organic constipation, being breast fed or fed a formula specially designed to treat constipation. Stool composition was measured by near-infrared reflectance analysis (NIRA) and parents answered questions about crying associated with defecation and stool consistency at baseline and after two weeks of the adapted formula.

**Results:**

After 2 weeks of the adapted formula, stool water content increased from 71 +/- 8.1% to 84 +/- 5.9%, (p < 0.02). There was no significant change in the stool's fat, protein or carbohydrate content. Parental impressions of constipation were improved with the decrease in stool hardness (100% with hard stools at baseline, 10% after 2 weeks), pain with defecation (90% at baseline, 10% after 2 weeks), and the requirement for rectal stimulation to achieve defecation (70% at baseline, 30% after 2 weeks, p < 0.001 for all three indicators).

**Conclusions:**

This preliminary study suggests that an adapted formula with high levels of lactose and magnesium increases stool water content and improves symptoms of constipation in term-born, formula-fed infants. A larger randomized placebo-controlled trial is indicated.

## Background

Constipation is usually defined in terms of changes in the frequency, size, and consistency of stools or difficulty in defecating [1]. It is a common cause of consultation, and in an observational study of 3487 infants, its prevalence was 7.8% (95 CI 6.9-8.7) [2]. Fewer bowel movements and/or hard stools may result in distension of the colon, pain, irritability and crying in the infant and this may be a cause of infantile colic [3,4]. Chronic constipation is a source of anxiety for parents who worry that a serious disease may cause this symptom [5]. The term "functional constipation" is used when no specific organic cause can be found. For younger infants, laxatives are not used and few infant formulae have been designed to solve the problem and for maintenance therapy.

Near-infrared reflectance analysis (NIRA) is a simple, rapid, reliable method to measure fat, protein, carbohydrate and water content in stools and has been used for over a decade [6-9]. In adults, it has been demonstrated that reduced stool weights in constipated subjects are linked to a reduction in stool water content [10].

The objective of this study is to analyze stool composition and parental assessment of symptoms of constipation with an adapted anti-constipation formula designed to reduce hard stools.

## Patients and method

### Patients

In the present study, constipation was characterized by the elimination of hard stools and by at least one of the following characteristics: excessive crying at the time of defecation (painful defecation), difficult passage or the need for external help to defecate [11].

Thirty constipated (according to the aforementioned definition) formula-fed infants aged 4-10 weeks were included. No organic cause of constipation was found in any of them. All were fed standard formula prior to enrolment and visited their paediatrician for constipation. All infants were full-term and had a normal birth weight (> 3.100 kg); none had a relevant medical history or pathological neonatal conditions. Preterm and low birth-weight infants were excluded. None were receiving an infant formula specially composed of modified nutrients or laxatives to treat constipation.

After enrolment, all 30 infants were fed Novalac AE (IT) (United Pharmaceuticals SA, France) for 2 weeks. The composition of this formula (Table 1) has been adapted in compliance with EU regulations to ease constipation [12,13]. Stools at baseline and after 2 weeks of the adapted formula were assessed for faecal fat, water, carbohydrates, and protein by NIRA. A questionnaire about crying during defecation, stool consistency and need for help with defecation was filled out by the parents at inclusion and after 2 weeks of the adapted formula. To evaluate stool consistency, the photographic Bristol stool form scale was used: stool hardness is classified from 1 to 7 according to the stool's appearance, in which 1-2 corresponds to hard stools ("like nuts, lumpy"), type 3 and 4 to intermediate, type 5-6 to soft stools and type 7 to completely liquid stools [14].

**Table 1:** Novalac AE (IT) composition

	Novalac AE Composition		EU regulation* (per 100 kcal)	
For	100 ml	100kcal	Min	Max
**Proteins (g)**	1,7	2,4	2,25	3
**Fats (g)**	3,5	5	3,3	6,5
Linoleic acid (g)	0,6	0,8	0,3	1,2
a-linolenic acid (mg)	56,4	79,8		
Palmitic acid(g)	0,78	1,2		
**Carbohydrates (g)**	8,1	11,4	7	14
Lactose	8,1	11,4	3,5	
**Energy (kcal)**	70,7	100	60	75
**(kJ)**	295.6	418,0		
**Minerals**				
Calcium (mg)	70	99	50	
Phosphorus (mg)	35,7	50,5	25	90
Calcium/Phosphorus	2			
Magnesium (mg)	9,1	12,9	5	15

* EU Directive 1991/321 CE on infant & follow-on formula, in force when the study was performed.

The study was coordinated and conducted by the Gastroenterology unit at Vall d'Hebron Children's Hospital (Barcelona, Spain) and the protocol was approved by the hospital's medical ethics committee (Comitè Ètic Hospital Vall d'Hebron). All parents granted their informed consent to participate in the study.

### Methods

The determination of fat, nitrogen, water and carbohydrates in the faeces was done using NIRA (near-infrared reflectance analysis) [6-9]. The NIRA technique is based on measuring radiation in the near-infrared spectrum (FENIR 8820 infrared analyser, Perten, Hamburg, Germany) on the surface of the sample, i.e. the matrix/substrate ratio, and the infrared reflection on the faecal surface at a given wavelength. The components measured belong to certain functional groups (CH, NH, OH) with specific absorption bands in the near-infrared spectrum (700-2500 nm). The spectroscopic response (reflection) of a faecal sample is thus related to the concentration of the components (functional groups). The correlation of the reflection taken at 12 wavelengths is determined using a computerised algorithm.

Prior to the current study, we compared NIRA measurements of stool fat, nitrogen, water and carbohydrate content in eighty stool samples from infants and children to the same measurements performed using traditional methods [6,8,15-17]. The normal values based on our own results, which are comparable to results from other authors, expressed as % (g/100 g of stools) are: fat: < 5%, nitrogen: < 1.8%, carbohydrates: < 2% and water: between 80 and 85%. However, the fat content for formula-fed infants is usually higher during the first 6 months of life (between 7 and 11%) [18,19].

### Statistical analysis

Non-parametric tests were used to test for changes in quantitative measurements over time (Wilcoxon sign-rank test) and categorical changes over time (McNemar test). The statistical analysis was performed using the SPSS 18.0 program (SPSS Inc, Chicago, IL).

## Results

### Clinical data

The results of the NIRA analysis are presented in table 2. Water content increased significantly (71% at inclusion, 84% after 2 weeks, p < 0.01). Other parameters (fat, nitrogen, carbohydrates) did not change.

**Table 2:** Stool NIRA at inclusion and follow-up (2 weeks).

NIRA	Normal values	Inclusion	After 2 weeks	P value*
Fat	7-11 (under 6 months) < 5 (over 6 months)	9.12 ± 3	8.35 ± 2.1	NS
Nitrogen	< 1.8	1.83 ± 0. 24	1.75 ± 0.22	NS
Carbohydrates	< 2	2 ± 0.94	1.96 ± 0.76	NS
Water	80-85	71 ± 8	84 ± 5.93	< 0.01

Values are expressed as % (wt/wt of stools).

* Wilcoxon Sign-rank test

For all infants, stools were reported as hard at inclusion (Bristol stool form scale type 1 in 23 infants and type 2 in 7 infants.). Defecation was described as painful (crying, irritability) in 90% of them and 70% required help to defecate. At the end of the follow-up period, stools had a soft consistency in 90% of the infants (Bristol stool form scale type 5 in 15 infants and type 6 in 12 infants) (p < 0.0001), 90% presented no pain or discomfort (p < 0.0001) and 70% did not require help to defecate (p < 0.001) (see Table 3).

**Table 3:** Parental assessments

		Inclusion	After 2 weeks	p value *
Stool consistency	Hard Soft	100%	10% 90%	<0.0001
Pain or difficulty	Yes No	90% 10%	10% 90%	<0.0001
External help need	Yes No	70% 30%	30% 70%	< 0,001

*McNemar's test

## Discussion

Breast-fed infants have significantly more stools per day than formula-fed ones and only a few have hard stools (1.1% of exclusively breast-fed infants vs. 9.2% of formula-fed infants [20,21]). The frequency of painful defecation in constipated children varies from 89% [22] to 40-50% [23,24] depending on the series reported. Fewer bowel movements due to prolonged transit time may result in distension of the gut, irritability and crying, and this may be a reason for inclusion in the associated group of infantile colic [2-4]. Tunc et al. report that, in the first 2 months of life, the median number of stools is lower in colicky infants than in those who do not have colic (p = 0.0001) [18].

Several hypotheses have been proposed to explain the softer stools associated with breast feeding. First, increased levels of gastric inhibitory polypeptide, motilin, neurotensin, vasoactive intestinal peptide secretions in formula-fed infants compared to breast-fed infants may explain their slower intestinal transit [25]. Second, more frequent breast feedings may stimulate the gastro-colic reflex resulting in more frequent defecation. Third, human milk contains large amounts of prebiotic oligosaccharides. Fourth, fat composition may play a role in softer stools. In breast milk and in most infant formulae, fats represent nearly 50% of energy content. The main saturated fatty acid in breast milk is palmitic acid (C16:0), with more than 70% esterified to the sn-2 position, whereas in regular infant formulae 88-94% of the palmitic acid is esterified to the sn-1,3 position. Lipolysis at the sn-1 and sn-3 positions requires pancreatic lipase, which is deficient in the first 6 months of life. The result is relative fat malabsorption, which may react with luminal calcium to form calcium soaps producing hard stools [25,26].

Previous NIRA studies have shown lower stool fat in breast-fed infants: 2.6% in breast-fed infants versus 7-11% in formula-fed infants younger than 6 months and less than 5% in formula-fed infants older than 6 months [9,10]. Our study confirmed the relative steatorrhoea in formula-fed infants [18,19]. Feeding a formula containing a high concentration of sn-2 palmitic acid caused softer stools but no change in stool frequency in one study [19] and decreased fatty acid soaps in another study [27]. We saw no change in stool fat content in the current study, suggesting that the improvement in constipation was not related to changes in fat metabolism.

A clinical trial by Chao et al. of 93 infants (average age 3.8 months) showed that the same formula we used in the current study (Novalac AE/IT) improved infant constipation. The infants receiving this formula had increased stool weight and decreased abdominal distension and irritability compared to infants receiving placebo [28]. Our study adds to these results and includes objective NIRA data.

The two additives to the adapted formula likely impact stool water content through different mechanisms. Lactose has prebiotic effects in that it is not completely absorbed in young infants and stimulates the growth of commensal bacteria. Non-hydrolyzed lactose reaches the colon, where it is metabolized by anaerobic microorganisms, producing an osmotic laxative effect as it attracts water into the intestinal lumen [29-32]. Magnesium, due to its osmotic properties, increases the laxative effect and stimulates intestinal motility by inducing cholecystokinin secretion [33].

In conclusion, this non-randomized, non-placebo-controlled preliminary study demonstrates that the use of an infant formula with high lactose and magnesium concentrations may increase stool hydration, which softens the stools with a corresponding clinical improvement. Thus, constipated infants who present hard stools may benefit from a change from standard formula to this specifically adapted formula. Larger randomized clinical trials on the efficacy of this formula are needed.

## Competing interests

The authors declare no competing interests.

## Authors' contributions

DI: designed, performed the research, analysed the data and wrote the paper. OS: performed the research. SR: performed the research. MA: performed the research. MM: revised data and wrote the paper. All authors read and approved the final manuscript.
